# Systems Immunology Analysis Reveals the Contribution of Pulmonary and Extrapulmonary Tissues to the Immunopathogenesis of Severe COVID-19 Patients

**DOI:** 10.3389/fimmu.2021.595150

**Published:** 2021-06-28

**Authors:** Sarah Musa Hammoudeh, Arabella Musa Hammoudeh, Poorna Manasa Bhamidimarri, Habiba Al Safar, Bassam Mahboub, Axel Künstner, Hauke Busch, Rabih Halwani, Qutayba Hamid, Mohamed Rahmani, Rifat Hamoudi

**Affiliations:** ^1^ Sharjah Institute for Medical Research, College of Medicine, University of Sharjah, Sharjah, United Arab Emirates; ^2^ Luebeck Institute of Experimental Dermatology, University of Luebeck, Luebeck, Germany; ^3^ General Surgery Department, Tawam Hospital, SEHA, Al Ain, United Arab Emirates; ^4^ Center for Biotechnology, Khalifa University of Science and Technology, Abu Dhabi, United Arab Emirates; ^5^ Department of Genetics and Molecular Biology, Khalifa University of Science and Technology, Abu Dhabi, United Arab Emirates; ^6^ Department of Respiratory Medicine, Rashid Hospital, Dubai Health Authority, Dubai, United Arab Emirates; ^7^ Meakins-Christie Laboratories, McGill University, Montreal, QC, Canada; ^8^ Division of Surgery and Interventional Science, University College London, London, United Kingdom

**Keywords:** COVID19, SARS-CoV-2, cytokine storm, immunopathogenesis, extrapulmonary tissues, liver, kidney, heart

## Abstract

As one of the current global health conundrums, COVID-19 pandemic caused a dramatic increase of cases exceeding 79 million and 1.7 million deaths worldwide. Severe presentation of COVID-19 is characterized by cytokine storm and chronic inflammation resulting in multi-organ dysfunction. Currently, it is unclear whether extrapulmonary tissues contribute to the cytokine storm mediated-disease exacerbation. In this study, we applied systems immunology analysis to investigate the immunomodulatory effects of SARS-CoV-2 infection in lung, liver, kidney, and heart tissues and the potential contribution of these tissues to cytokines production. Notably, genes associated with neutrophil-mediated immune response (e.g. CXCL1) were particularly upregulated in lung, whereas genes associated with eosinophil-mediated immune response (e.g. CCL11) were particularly upregulated in heart tissue. In contrast, immune responses mediated by monocytes, dendritic cells, T-cells and B-cells were almost similarly dysregulated in all tissue types. Focused analysis of 14 cytokines classically upregulated in COVID-19 patients revealed that only some of these cytokines are dysregulated in lung tissue, whereas the other cytokines are upregulated in extrapulmonary tissues (e.g. IL6 and IL2RA). Investigations of potential mechanisms by which SARS-CoV-2 modulates the immune response and cytokine production revealed a marked dysregulation of NF-κB signaling particularly CBM complex and the NF-κB inhibitor BCL3. Moreover, overexpression of mucin family genes (e.g. MUC3A, MUC4, MUC5B, MUC16, and MUC17) and HSP90AB1 suggest that the exacerbated inflammation activated pulmonary and extrapulmonary tissues remodeling. In addition, we identified multiple sets of immune response associated genes upregulated in a tissue-specific manner (DCLRE1C, CHI3L1, and PARP14 in lung; APOA4, NFASC, WIPF3, and CD34 in liver; LILRA5, ISG20, S100A12, and HLX in kidney; and ASS1 and PTPN1 in heart). Altogether, these findings suggest that the cytokines storm triggered by SARS-CoV-2 infection is potentially the result of dysregulated cytokine production by inflamed pulmonary and extrapulmonary (e.g. liver, kidney, and heart) tissues.

## Introduction

The outbreak of Coronavirus disease 2019 (COVID-19) pandemic caused by the severe acute respiratory syndrome coronavirus 2 (SARS-CoV-2), led to more than 79 million cases and 1.7 million deaths worldwide to date, as reported by the World Health Organization. The clinical presentation of COVID-19 is characterized by a wide variation in severity and symptoms reflecting both pulmonary and extra-pulmonary manifestations including hepatic and renal dysfunction and heart failure (1, 2). Importantly, expression of angiotensin converting enzyme II (ACE2), which is implicated in the cellular entry of SARS-CoV-2, in various extrapulmonary human tissues including proximal tubular cells (kidney), cholangiocytes (liver), and myocardial cells (heart) suggested the vulnerability of these tissues to SARS-CoV-2 infection (3). Moreover, SARS-CoV-2 infectivity of extrapulmonary tissues including liver, kidney, and heart tissues has been proven using *in vitro* cultures, organoid models, and tissue autopsies (4–6).

One of the critical mechanisms contributing to the progression of COVID-19 is the viral induction of cytokine storm, a phenomenon characterized by the unrestrained production and release of cytokines into the circulation resulting in a systemic inflammation (7). This uncontrolled release of cytokines dysregulates innate and adaptive immune responses, resulting in the infiltration of various immune effectors into different tissues. In this regards, analysis of pulmonary and extrapulmonary tissue biopsy/autopsy samples revealed interstitial infiltration of lymphocytes, macrophages, neutrophils, NK cells and dendritic cells into lung tissue (8, 9); lymphocytes into liver tissue (10); lymphocytes and macrophages into kidney tissue (11); and mononuclear inflammatory cells into heart tissue (8). Although these immune cells are initially attracted to counteract SARS-CoV-2 infection, the exacerbation of the immune response elicited by the overexpressed cytokines and transcriptional shifts can potentially promote tissue remodeling, cellular apoptosis and tissue dysfunction and cytotoxicity (11). Moreover, the progression of systemic inflammation and cytokine storm (e.g. significantly increased IL-2, IL-7, IL-10, GSCF, IP10, MCP-1, MIP1A and TNF-α levels) can potentially lead to multi-organ dysfunction (observed in approximately 5% of COVID-19 patients) and viral sepsis increasing the risk of mortality (12, 13).

This study aimed at comparing changes in immune response activation patterns across pulmonary and extrapulmonary tissues (i.e. liver, kidney, and heart) in response to SARS-CoV-2 infection. Therefore, The differential immune transcriptomic profile of lung, liver, kidney, and heart autopsy samples from COVID-19 patients was compared.

## Materials and Methods

### RNA-Seq Datasets Retrieval

Datasets were retrieved from publicly available sets deposited in Gene Expression Omnibus (GEO) for Covid-19 infected lung, liver, kidney, and heart samples from 12 patient autopsies (GSE150316) (a total number of 58 COVID-19 samples from all tissues), and healthy lung, liver, kidney, and heart autopsy samples (GSE112356). 6 liver samples (cases 3, 4, 5, 8, 10, 12), 3 kidney samples (cases 4, 5, and 11), 7 heart samples (cases 1, 2, 3, 4, 5, and 8), and 42 lung samples (cases 1, 2, 3, 4, 5, 6, 7, 8, 9, 10, and 11) were selected from the COVID-19 patient autopsy dataset (GSE150316) for further analysis (**Table 1**). As elaborated in **Table 1**, multiple samples were taken from different locations in lung (case1: 4 samples, case 2: 3 samples, case 3: 2 samples, case 4: 2 samples, case 5: 5 samples, case 6: 5 samples, case 7: 5 samples, case 8: 5 samples, case 9: 5 samples, 3 from case 10, and 3 from case 11) and heart tissues; however, all samples were taken into consideration to avoid biased selection.

**Table 1:** List of analyzed healthy and COVID-19 autopsy samples retrieved from the datasets GSE112356 and GSE150316 deposited in GEO.

Tissue Source	Healthy Autopsy Samples	COVID-19 Autopsy Samples	Total number of analysed samples
Lung	H1Lu; H2Lu; H3Lu; H4Lu	case1: samples 1, 2, 3, 4 case2: samples 1, 2, 3 case3: samples 1, 2 case4: samples 1, 2 case5: samples 1, 2, 3, 4, 5 case6: samples 1, 2, 3, 4, 5 case7: samples 1, 2, 3, 4, 5 case8: samples 1, 2, 3, 4, 5 case9: samples 2, 3, 4, 5 case10: samples 1, 2, 3 case11: samples 1, 2, 3	4 healthy samples and 42 COVID19 samples
Liver	H1Li; H2Li; H3Li; H4Li	case3: sample 1 case4: sample 1 case5: sample 1 case8: sample 1 case10: sample 1 case12: sample1	4 healthy samples and 6 COVID19 samples
Kidney	H1K; H2K; H3K; H4K	case4: sample 1 case5: sample 1 case11: sample 1	4 healthy samples and 3 COVID19 samples
Heart	H2H; H3H; H4H	case1: sample 1 case2: sample 1 case3: sample 1 case4: samples 1, 2 case5: sample 1 case8: sample 1	3 healthy samples and 7 COVID19 samples

As reported by the authors depositing the dataset (14), the donors of the COVID-19 samples were severe COVID-19 patients; the majority requiring mechanical ventilation during their hospitalization (9 out of 12) and were deceased after 6-23 days of hospitalization, with a 100% mortality rate. Some of the donors were characterized to be immunocompromised (cases 1, 3, 6, 9, 11) while others were diabetic (cases 1, 3, 4, 5, 6, 12) and were under anti-diabetic and immunosuppressive treatments prior to contacting SARS-CoV-2 infection. The majority of the patients were reported to have elevated levels of D-Dimer (Cases 1-10 and 12), CRP (Cases 1-10 and 12), and AST (Cases 2-10 and 12), which were proposed to correlate with COVID-19 severity, inflammation and extrapulmonary tissue dysfunction (15, 16).

15 healthy autopsy samples were selected from the dataset GSE112356 including 4 healthy lung samples, 4 healthy liver samples, 4 healthy kidney samples and 3 healthy heart samples (excluding the sample H1H as it was reported to be misclassified) collected from Caucasian male donors.

Whole blood bulk RNA sequencing data (Normalized counts) deposited by Bernardes et al. (17) were retrieved for the cross validation of the autopsy tissue samples analysis (https://github.com/Systems-Immunology-IKMB/COVIDOMICs/tree/main/TF_enrichment/TF_enrichment_analysis-main/data). The dataset included samples from 14 healthy donors, 12 asymptomatic COVID-19 patients, 11 mild, 19 severe (subdivided into 6 complicated, 4 complicated incremental, 6 complicated hyperinflammatory, and 3 critical) COVID-19 patients. Complicated-incremental refers to severe COVID-19 patients with increasing clinical symptoms and inflammatory markers; Complicated-hyperinflammatory refers to patients presenting with severe signs of systemic inflammatory response; and critical refers to patients requiring ICU administration and mechanical ventilation with signs of acute respiratory distress.

### Power Calculation

To determine if the cohort used in this analysis has sufficient power to allow for the identification of predictive biomarkers, power calculation based on Wei et al. (18) was carried out. Since there are limited phenotypic data on severe COVID19 cases with affected extrapulmonary tissues in response to SARS-CoV-2 infection, well characterized severe COVID19 cases mainly from autopsy samples were included for biomarker discovery. Thus, based on whole transcriptome data of severe COVID19 studies, the standard deviation for detection of differentially expressed genes for severe cases across different tissue was determined to be around 1.5 (σ = 1.5) and effect size around 5 in well characterized cases. Carrying out the power calculation at p = 0.05 (5% significance testing) and power of 90% using R (version 3.6.2) showed that the minimum required number of samples per group to be 3 patients per group for each tissue (healthy tissue autopsy vs. lung, liver, kidney and heart tissue from severe COVID-19 patient autopsies) or disease severity group (healthy, mild, moderate and severe). Having said that, the biomarkers discovered were validated on a larger cohort of whole blood samples from different groups of COVID-19 patients.

### Bioinformatics Analysis and Identification of Differentially Expressed Genes

Raw gene counts data were retrieved for all the samples and the successfully mapped genes overlapping between the samples from both datasets were filtered for further analysis (21831 overlapping genes). The row gene counts were then normalized (quantile normalization) using AltAnalyze software (19). Differentially expressed genes (DEGs) were identified by comparing each SARS-CoV-2 infected tissue against a healthy control tissue. Cutoff values for DEGs included fold change value >2 or <-2 and adjusted p-value (q-value) of 0.25 based on the methodologies described by Li et al. (20) and Subramanian et al. (21), where they chose < 0.25 cutoff for adjusted p-value to select for DEG and pathways. DEGs were intersected to identify the commonly upregulated and downregulated genes between the infected lung, liver and kidney tissues using InteractiVenn (22).

### *In Silico* Functional Analysis

Immune response genes were identified from the DEGs using immune response gene ontology set (GO:0006955) as a reference, which were then further analyzed for functional clustering and pathway enrichment using Metascape (23). To identify the specific effect of SARS-CoV-2 infection on the expression of immune response, we cross matched the differential transcriptome from the infected lung, liver, and kidney samples with gene ontology sets retrieved using AmiGO 2 database (**Table S1**). Heatmap and dotplot representations were generated using R (version 3.6.0); histogram representations were generated using GraphPad Prism (version 5.01).

### Quantitative Real-Time PCR Analysis of Gene Expression

Blood Serum was isolated from fresh blood samples collected from 3 healthy donors, 6 asymptomatic COVID-19 patients, 2 severe COVID-19 patients with pulmonary findings only, and 4 severe COVID-19 patients with pulmonary and extrapulmonary findings (i.e. elevated levels of creatinine and liver enzymes) following the approval of the ethical committee at by the Abu Dhabi Health COVID-19 Research Ethics Committee (DOH/DQD/2020/538), SEHA Research Ethics committee (SEHA-IRB-005) and Dubai Scientific Research Ethics Committee (DSREC-04/2020_09). The blood plasma was isolated using histopaque gradient separation (Sigma). Total RNA was extracted from 300µl of Plasma using QIAamp Viral RNA Mini Kit (Qiagen). cDNA was synthesized using the High-Capacity cDNA Reverse Transcription Kit for RT PCR (Applied Biosystems). qRT-PCR was performed in triplicates with the Maxima SYBR Green/ROX qPCR Master Mix (Thermoscientific) using QuantStudio3 Real-Time PCR instrument (Applied biosystems). qRT-PCR were performed using primers for 18SrRNA, SOCS3, and TRIM56 as per the sequences in **Table S2**.

### Statistical Analysis

Unpaired, two-tailed t-test statistical analysis was performed using GraphPad Prism (version 5.01) to analyze the statistical significance of the gene expression. The significance was taken to be p < 0.05.

## Results

### General Effect of SARS-CoV-2 Infection on the Transcriptomic Profile of Lung, Liver, Kidney, and Heart Tissues

Analysis of the general effect of SARS-CoV-2 infection of the transcriptomic profiles of pulmonary and extrapulmonary tissues (i.e. liver, kidney, and heart tissue) showed the upregulation of 3421 genes in lung tissue, 4805 genes in liver tissue, 5685 genes in kidney tissue, and 4176 genes in heart tissue (**Figure S1A**). Out of these differentially upregulated genes, 1537 genes were commonly upregulated across the four types of tissues (**Figure S1B**). Functional clustering analysis revealed the implication of these transcripts in viral infection, intracellular and across membrane transport, GPCR signaling, WNT and mTOR signaling, tissue development and morphogenesis, response to transforming growth factor beta signaling, regulation of protein modification and degradation, response to granulocyte macrophage colony-stimulating factor, and response to hormonal stimulus and growth factors (**Figure S1C**).

Moreover, the analysis revealed the downregulation of 3928 genes in lung tissue, 3881 genes in liver tissue, 4848 genes in kidney tissue, and 3312 genes in heart tissue. 1375 genes out of these genes were commonly downregulated across the four types of tissues (**Figure S1D**). Most of the commonly downregulated transcriptome were shown to be involved in the regulation of metabolic pathways, protein translation, mitochondrial processes, and autophagy through functional clustering analysis (**Figure S1E**).

### SARS-CoV-2 Infection Induces a Greater Immunomodulatory Effect on Liver and Kidney Tissues in Comparison to Lung and Heart Tissue

**Figure 1:**
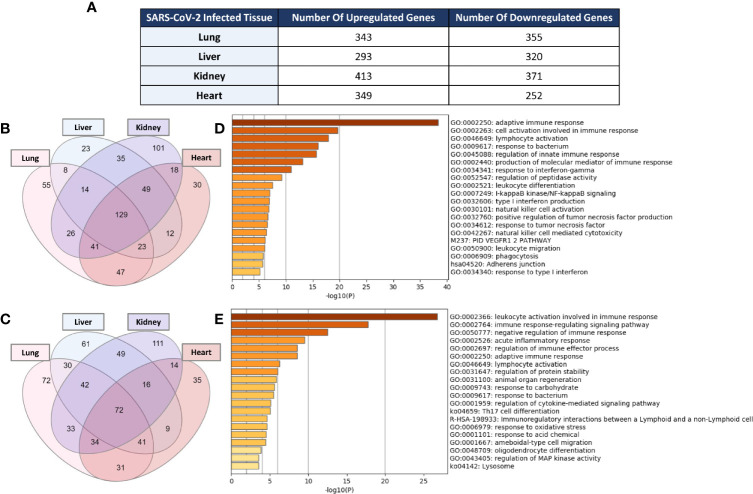
**(A)** Count of differentially expressed immune response genes from the immune response gene ontology set (GO:0006955) in SARS-CoV-2 infected lung, liver, kidney, and heart tissues. Venn diagram representation of the overlap of significantly **(B)** upregulated and **(C)** downregulated immune response genes amongst the four types of infected tissues. Functional clustering and pathway analysis of the commonly **(D)** upregulated and **(E)** downregulated genes across lung, liver, kidney, and heart tissues in response to SARS-CoV-2 infection.

Examination of the enrichment of differentially expressed immune response genes in SARS-CoV-2 infected lung, liver, kidney, and heart tissues was carried out using the immune response gene ontology set (GO:0006955) from AmiGO 2 database as a reference. The count of the differentially expressed genes across the four types of infected tissues were then compared (**Figure 1A**). Intriguingly, the count of the immune response genes differentially expressed in severe COVID-19 patients’ liver, kidney, and heart tissue was relatively close to that of the genes differentially expressed in lung tissue. 129 immune response genes were commonly upregulated amongst the four types of tissue in response to severe SARS-CoV-2 infection (**Figure 1B**) while 72 genes were commonly downregulated (**Figure 1C**).

Functional clustering and pathways analysis of the differentially expressed genes common to the four different types of infected tissues revealed a significant enrichment of transcripts implicated in many arms of the innate and adaptive immune response including the activation, chemotaxis and differentiation of leukocytes; cytokines production and signaling; response to interferon-gamma; cell-cell adhesion; NF-κB signaling; and acute inflammatory response (**Figures 1D, E**).

### Effects of SARS-CoV-2 Infection on Expression of Cytokine Storm Related Genes in Pulmonary and Extrapulmonary Tissues

**Figure 2:**
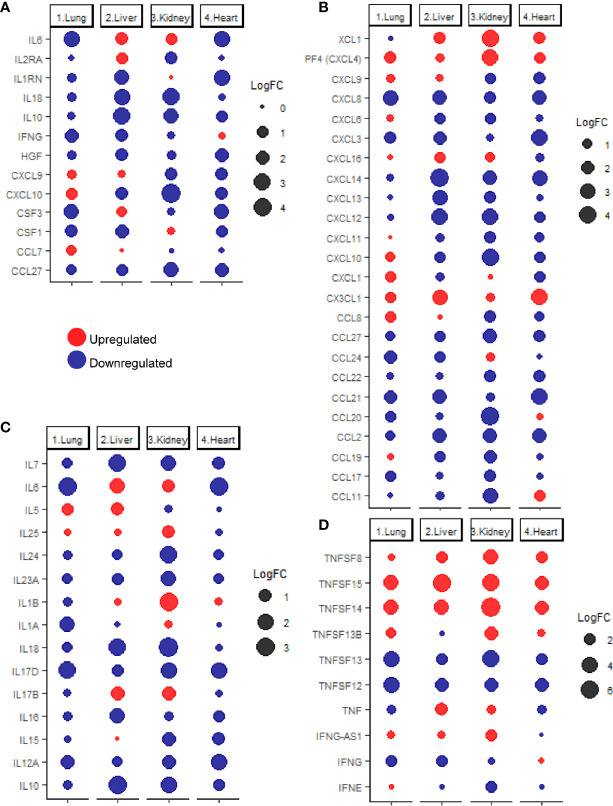
**(A)** dotplot representation of the expression fold change of cytokines contributing to the cytokine storm in response to SARS-CoV-2 infection in lung, liver, kidney, and heart tissues. Dotplot representation of the differentially expressed **(B)** chemokines, **(C)** interleukins, **(D)** interferons and tumor necrosis factor family members in lung, liver, kidney, and heart tissues infected with SARS-CoV-2.

Previous studies reported that COVID-19 is frequently associated with a massive production of 14 cytokines including IFN-γ, IL-1RA (IL1RN), IL-2RA, IL-6, IL-10, IL-18, HGF, MCP-3 (CCL7), MIG (CXCL9), M-CSF (CSF1), G-CSF (CSF3), MIG-1a (CCL3), CTACK (CCL27), and IP-10 (CXCL10) (24). Therefore, the next aim of the study was to investigate the expression levels of these cytokines in pulmonary and extrapulmonary tissues such as heart, Kidney, and liver. As shown in **Figure 2A**, out of the 14 cytokines analyzed, 4 cytokines were differentially expressed (downregulated) in lung tissue including *IL6, CSF3, IFNG*, and *CSF1*. 10 of the cytokines were differentially expressed in liver tissue: IL6 and IL2RA were upregulated and *IL10, IL18, IL1RN, CSF1, IFNG, CXCL10, HGF*, and *CCL27* were downregulated. A total of 7 of the cytokines were differentially expressed in kidney tissue, out of which 1 cytokine was upregulated (IL6) and 6 cytokines were downregulated (*CXCL10, IL18, CCL27, IL10, IL2RA*, and *CXCL9*). Eight cytokines were differentially expressed (downregulated) in heart tissue including *IL6, IL1RN, CSF3, HGF, CCL27, CXCL9, CXCL10*, and *CSF1*.

Although some of the cytokines were commonly, differentially expressed in pulmonary and some extrapulmonary tissues (e.g. *IL6, IFNG, CSF3*, and *CSF1*), the differential expression of some of the cytokines is limited to extrapulmonary tissues such including *IL2RA, IL1RN, IL18, IL10,* and *HGF*, and *CXCL9* (**Figure 2A**). Altogether, these results suggest that the uncontrolled production of cytokines induced by SARS-CoV-2 infection is potentially contributed by pulmonary and extrapulmonary tissues.

### SARS-CoV-2 Infection Causes Shifts in the Gene Expression of Cytokines in Various Tissues

Further evaluation of changes in the expression of extended list of cytokines across lung, liver, kidney, and heart tissue was carried out using the chemokine gene set within the immunome gene ontology set as a reference. Analysis of the differentially expressed chemokines (**Figure 2B**) revealed the unique upregulation of the neutrophil specific chemokine *CXCL1* in lung (25), and the eosinophil specific chemoattractant *CCL11* in heart tissue (26). Monocytes chemoattractants were enriched in lung (*CXCL4, CCL8*, and *CX3CL1)*, liver, (*CX3CL1*), kidney (*CXCL4*), and heart (*CXCL4*, and *CX3CL1*) (27, 28). Dendritic cells chemoattractants were enriched in lung (*CCL8* and *CX3CL1*), liver (*XCL1* and *CX3CL1*), kidney (*XCL1*), and heart (*XCL1* and *CX3CL1*) (29–33). T-cells and B-cells chemotactic chemokines were upregulated in lung (*CCL8, CXCL4* and *CX3CL1*), liver (*CXCL16*), kidney (*CXCL4* and *CXCL16*) and heart (*CXCL4* and *CX3CL1*) (29, 30, 32, 34).

Analysis of the differential expression of interleukins (**Figure 2C**) revealed the upregulation of eosinophil activator IL5 and activator of T helper cell immune response IL17B in liver and the activators of T helper cell immune response IL1B and IL17B as well as IL25 (activator of NKT cells, T helper cells, neutrophils, eosinophils, and mast cells) in kidney (35–38). On the other hand, none of the interleukins was significantly upregulated in lung or heart tissues.

Interferon gamma (IFNG) was significantly downregulated in infected lung and liver tissues (**Figure 2D**) while changes in the interferon alpha and beta production were negligible (FC <2 or >-2 or q-value>0.25). The absence of dysregulated interferon alpha and beta production has been previously reported in analysis of samples of COVID-19 patients which was proposed to contribute to the dysregulated early innate immune response (39, 40). interferon epsilon (IFNE) was differentially expressed (downregulated) in kidney.

Analysis of the differential expression of tumor necrosis factor family members revealed the upregulation of *TNFSF13B, TNFSF14*, and *TNFSF15* expression in lung; *TNF, TNFSF8, TNFSF14* and *TNFSF15* in liver; *TNFSF8, TNFSF13B, TNFSF45* and *TNFSF15* expression in kidney; and *TNFSF8, TNFSF14*, and *TNFSF15* in heart (**Figure 2D**). Tumor necrosis factor alpha (TNF) was only significantly upregulated in liver tissue in response to SARS-CoV-2 infection, indicating the potentially critical role of contributed by infected liver tissue in the exacerbation of systemic inflammation and cytokines storm (41).

### Functional Clustering and Pathway Analysis Reveals Enrichment of Genes Implicated in IL-6 and Interferon-Gamma Production in Pulmonary and Extrapulmonary Tissues

As the degradation of the mRNA can distort the expression results of the cytokines, the next aim was to cross-validated the cytokines response pathways using functional clustering and pathways analysis of differentially expressed immune response genes in SARS-CoV-2 infected tissues. Transcripts implicated in IL-6 production pathway (GO:0032635) was significantly enriched in the upregulated transcriptome of liver and kidney tissues (log10 q-value -9.11 and -8.93, respectively). On the other, analysis of the downregulated transcriptome revealed the enrichment of transcripts implicated in cellular response to IL-1 (GO:0071347) in lung, liver, kidney and heart tissues (log10 q-value -22.56, -19.22, -29.48, and -25.59, respectively).

Upregulated transcripts implicated in the response to interferon alpha and beta (Reactome gene set: R-HSA-909733) were enriched in lung and heart tissues (log10 q-value -10.69 and -6.94, respectively), whereas downregulated transcripts were more enriched in infected heart tissue (log10 q-value -10.03). Transcripts contributing to interferon gamma response (GO:0034341) were dysregulated in the upregulated transcriptome lung, liver, kidney, and heart tissues (log10 q-value -19.24, -13.90, -20.27, and -17.70, respectively), as well as in the downregulated transcriptome of kidney tissue (log10 q-value -20.86). Functional clustering and pathway analysis of the differentially expressed immune response genes revealed the upregulation of tumor necrosis factor superfamily cytokine production (GO:0071706) in liver and kidney tissues (log10 q-value -8.65 and -13.00, respectively). On the other hand, transcripts implicated in the response to tumor necrosis factor (GO:0071356) were significantly downregulated in lung, liver, kidney, and heart tissues (log10 q-value -23.47, -27.36, -26.20, and -30.08, respectively).

Altogether, these results suggest that both pulmonary and extrapulmonary tissues may play important role in the dysregulated levels of interleukins (e.g. IL-6 and IL-1), interferon-gamma, and tumor necrosis factor superfamily cytokines in COVID-19 patients.

### SARS-CoV-2 Infection Significantly Elicits Innate and Adaptive Immune Response in Pulmonary and Extrapulmonary Tissues

Contextualizing the differential expression of the identified cytokines as a part of the innate and adaptive immune responses was carried out by focusing the functional clustering and pathway analysis to assess the enrichment of the innate (GO:0002218) and adaptive (GO:0002250) immune response clusters in the differential transcriptome of each of the tissues.

In infected lung tissue, the innate and adaptive immune response functional clusters were significantly enriched in the upregulated transcriptome (log 10 q-value: -38.37 and -72.10) as well as in the downregulated transcriptome (-28.30 and -55.36), respectively. A similar pattern was observed in liver, kidney and heart tissue, revealing a significant enrichment of the innate immune response in the upregulated (-29.14, -34.20, and -31.84) and downregulated (-33.71, -49.14, and -32.08) transcriptomes; as well as a significant enrichment of the adaptive immune response in the upregulated (-80.25, -94.66, -81.95) and downregulated (-50.04, -46.11, and -37.06) transcriptomes, respectively. Remarkably, the pulmonary tissue presented a higher number of upregulated transcripts contributing to the innate immune response, whereas the extrapulmonary tissues revealed a higher number of upregulated transcripts contributing to adaptive immune response.

Taken together, these findings suggest that innate and adaptive immune response elements are significantly dysregulated in pulmonary and extrapulmonary tissues. However, infected lung tissue might display a relatively more inflammatory phenotype in response to the higher upregulation of the innate immune response genes in comparison to other tissues.

### Enrichment of Immune Effectors Activation and Chemotaxis Pathway Genes in Response to SARS-CoV-2 Infection in Pulmonary and Extrapulmonary Tissues

**Figure 3:**
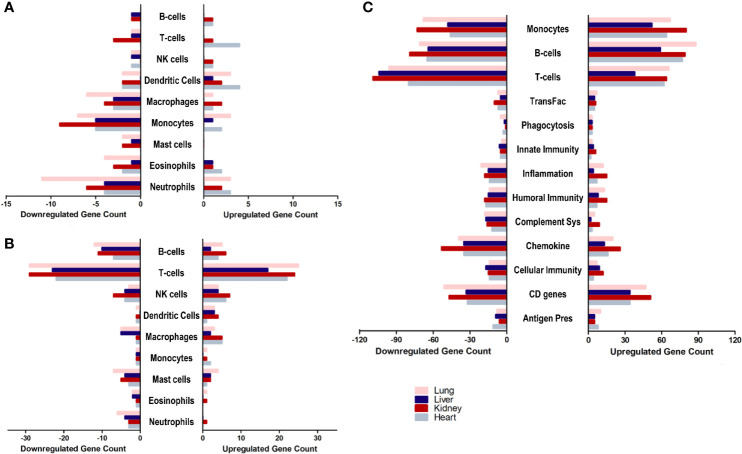
The count of differentially expressed genes in SARS-CoV-2 infected lung, liver, kidney, and heart tissues overlapping with the **(A)** immune cells chemotaxis gene ontology set, **(B)** immune cells activation gene ontology sets, and **(C)** Immunome gene ontology set ontology sets.

With a general idea about the immune response polarization for each of the tissues, the next aim was to further analyze the enrichment of transcripts implicated in the response of each immune effector. Therefore, the overlap of the differential transcriptome of SARS-CoV-2 infected lung, liver, kidney and heart tissues with immune cells chemotaxis gene ontology set (**Figure 3A**), immune cells activation gene ontology set (**Figure 3B**), and immunome gene ontology set (**Figure 3C**) was examined.

The analysis revealed a similar immunomodulatory effect of SARS-CoV-2 on extrapulmonary tissues (especially kidney tissue) to that exerted on the pulmonary tissue, which correlates with the close number of immune response genes differentially expressed across all examined tissues. However, a relatively higher immunomodulatory effect was observed on the extrapulmonary tissue in the chemotaxis of T-cells, B-cells, NK cells, and dendritic cells (**Figure 3A**) as well as the activation of NK cells, macrophages, and monocytes (**Figure 3B**). Moreover, kidney tissue was more enriched in transcripts implicated in inflammation, monocytes-mediated immune response, innate immunity, cytokines expression, and cellular immunity (**Figure 3C**).

These results support the speculation on the potential contribution of infected extrapulmonary tissues to the exacerbation of the dysregulation of the immune response and systemic inflammation, alongside infected pulmonary tissue in response to SARS-CoV-2 infection.

### SARS-CoV-2 Infection Modulates NF-κB Signaling and CBM Complex as a Potential Regulatory Mechanism of the Different Arms of Immune Response

**Figure 4:**
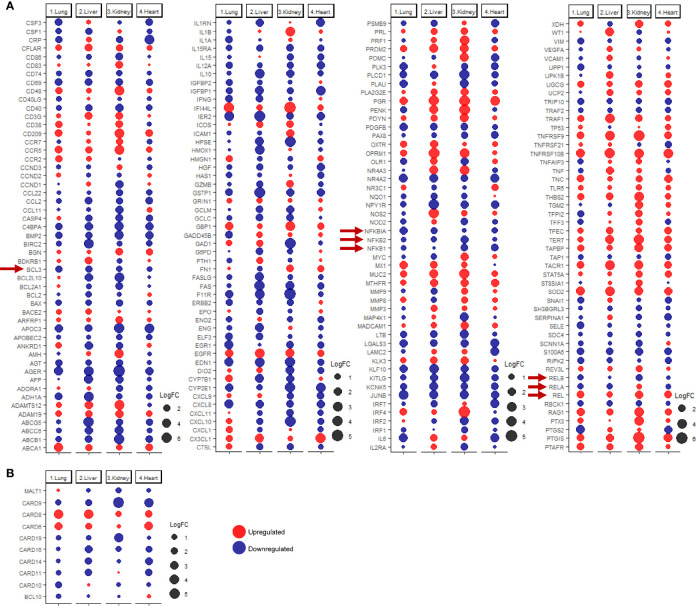
dotplot representation of the expression fold change of **(A)** inducible NF-κB genes and **(B)** CBM complex and CARD family proteins in lung, liver, kidney, and heart tissues infected with SARS-CoV-2. Red arrows representing core elements of the NF-κB signaling pathways.

Since many of the discussed immune response genes are inducible by NF-κB signaling (**Figure 4A**), the next aim of the study was to investigate the dysregulation of NF-κB signaling as a potential underlying cause for the observed immune transcriptomic shifts in pulmonary and extrapulmonary tissues. Analysis of the differential expression revealed a predominant pattern towards the dysregulation of the NF-κB pathway elements (e.g. *RELB*, *NFKB1*, *NFKB2*, and *BCL3*) (**Figure 4A**). Moreover, the expression of the CBM signalosome complex genes, *BCL10, MALT1* and CARD family members (*CARD6, CARD8, CARD9, CARD10, CARD11, CARD14*), which regulate and activate NF-κB signaling, was dysregulated across the four types of tissues (**Figure 4B**) (42–44).

Functional clustering and pathway analysis of the differentially expressed immune response genes revealed a significant enrichment of NK-κB signaling pathway genes (GO:0038061) in the differential transcriptome of the four types of tissue. The enrichment of NF-kB signaling elements was most significant in kidney, then heart, liver, and the least in lung tissue (log10 q-value -34.35, -29.38, -27.37, and -21.10, respectively).

These results suggest that SARS-CoV-2 infection induces a shift in the activation state of NF-κB signaling, resulting in the transcriptional shift of NF-κB inducible genes and aberrant production of cytokines.

### Analysis of Commonly and Uniquely Upregulated Immune Response Genes in Each of the Tissues in Response to SARS-CoV-2 Infection

**Figure 5:**
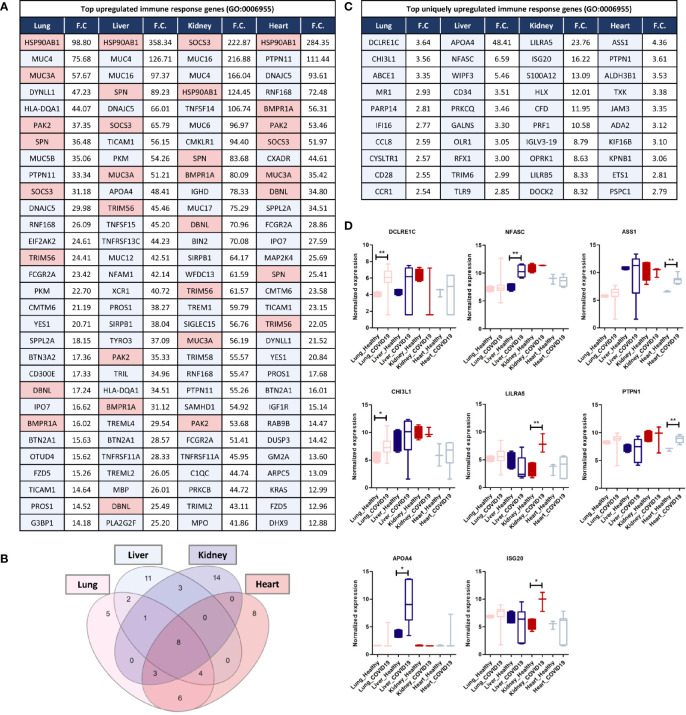
**(A)** Top 30 upregulated immune response genes in lung, liver, kidney, and heart tissues in response to SARS-CoV-2 infection; F.C.: fold change; light red represents commonly upregulated genes within the top 20 genes across the 4 types of tissues. **(B)** Venn diagram representation of the overlap between the top 20 upregulated immune response genes in each tissue, enlisted in **(A)**. **(C, D)** Top Immune response genes uniquely upregulated in lung, liver, kidney, and heart tissues in response to SARS-CoV-2 infection. * represents p-value < 0.05; ** represents p-value < 0.01 analyzed using T-test statistical analysis.

Analyzing commonly and uniquely differentially expressed genes across lung, liver, kidney, and heart tissue to identify potential diagnostic biomarkers for tissue-specific inflammation was carried out by examining the overlap amongst the top 20 immune response genes upregulated in each of the tissues in response to SARS-CoV-2 infection revealed 8 common genes including *HSP90AB1, MUC3A, PAK2, SPN, SOCS3, TRIM56, DBNL*, and *BMPR1A* (**Figures 5A, B**).

Tripartite motif 56 (TRIM56) is an important regulator of innate immunity and TLR3-mediated anti-viral defense against various infections including hepatitis C virus (45). Moreover, two of the genes commonly upregulated are the suppressor of cytokine signaling-3 (SOCS3) and the heat shock protein HSP90AB1, which contribute to the regulation of cytokines production and signaling and subsequently, immune response and inflammation (46, 47). Furthermore, the mucin family genes, including *MUC3A, MUC4, MUC5B, MUC16*, and *MUC17* were consistently upregulated across the four types of tissues (**Figure 5A**). LPCAT1, is another commonly upregulated gene between the four types of tissues, and it a regulator of inflammatory lipids synthesis (e.g. platelet-activating factor and lysophosphatidylcholine) (48).

Analysis of significantly and uniquely upregulated genes in each of the tissues in response to SARS-CoV-2 infection (**Figure 5**) showed that 55 genes were uniquely upregulated in lung tissue which included multiple important immune response regulators (e.g. *DCLRE1C, CHI3L1, and PARP14*) as well as cytokine receptors (e.g. *CCR1, CCR2*, and *IL1RL1*) (**Figures 5C, D**). Chitinase 3-like-1 (CHI3L1), was shown to play an important role in antipathogen innate and adaptive immune response in addition to apoptosis and tissue remodeling (49). poly-ADP-ribose polymerase (PARP14) similarly plays a central role in the activation and regulation of anti-viral interferon-based immune response (50).

23 immune response genes were significantly and uniquely upregulated in liver tissue which included some cytokines (e.g. TNFRSF4 and *CXCL16)* and anti-inflammatory proteins and immune checkpoints (e.g. *APOA4*) (**Figures 5C, D**); which could potentially indicate the activation of a negative feedback loop to regulate the aberrant expression of cytokines and immune mediators (51).

101 genes were significantly uniquely upregulated in kidney tissue which included some cytokines and cytokine receptors (e.g. *TGFB3, CXCR2, IL5RA*, and *IL36NA*) as well as multiple immune response regulators including the monocyte activator *S100A12* (52), the interferon production regulator H2.0-like homeobox 1 (*HLX)* (53)*, LILRA5*, and *ISG20* (52, 53) (**Figures 5C, D**). Moreover, interferon-induced protein 20 (ISG20), an antiviral protein that directly degrades viral RNA (54), was uniquely upregulated in kidney tissue. Moreover, The upregulation of adhesion regulatory molecules including the intercellular adhesion molecule 5 (ICAM5) (55) and Glia maturation factor gamma (GMFG; regulates T-cell chemotaxis by regulating adhesion molecular and cellular detachment) (56) in kidney supports the observed enrichment of leukocytes (especially T-cells) recruitment pathways and immune cells infiltration into tissue biopsies in response to SARS-CoV-2 infection (11).

30 genes were significantly uniquely upregulated in heart tissue which included antiviral and immune response regulators (e.g. ASS1 and PTPN1) (**Figures 5C, D**). ASS1 regulates cellular survival, autophagy, immune cells differentiation (57, 58). Protein tyrosine phosphatase 1B (PTPN1) is a regulator of anti-viral interferon-based innate immune response by mediating the dephosphorylation of the mediator of IRF3 activation (MITA/STING) and a facilitator of macrophage-mediated inflammation (59, 60).

### Cross-Validation of Immune Response Putative Biomarkers in COVID-19 Patients’ Whole Blood Samples

**Figure 6:**
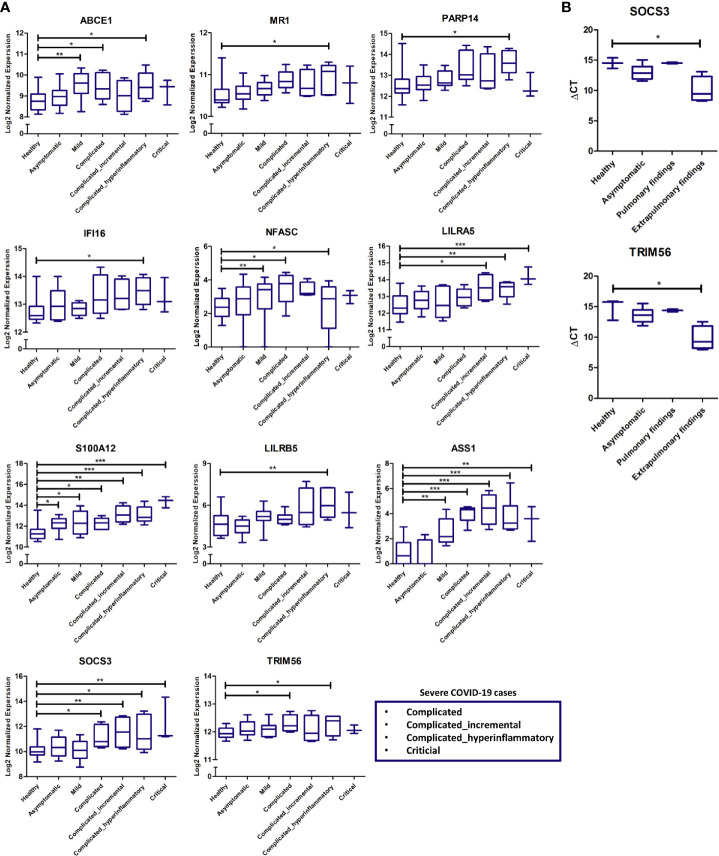
**(A)** Gene expression cross-validation of identified uniquely and commonly expressed immune putative biomarkers across pulmonary and extrapulmonary tissues using RNA sequencing dataset of whole blood samples from healthy donors and asymptomatic, mild, and severe COVID-19 patients. **(B)** Gene expression cross-validation of commonly expressed immune putative biomarkers across pulmonary and extrapulmonary tissues using qRT-PCR analysis of blood plasma samples from 3 healthy donors as well as 6 asymptomatic COVID-19 patients, 2 severe COVID-19 patients with pulmonary findings (pulmonary findings) only, and 4 severe COVID-19 with pulmonary and extrapulmonary finding (extrapulmonary findings). * represents p-value < 0.05; ** represents p-value < 0.01; *** represents p-value < 0.001; analyzed using unpaired t-test statistical analysis.

Validation of some of the putative biomarkers from the discovery analysis shown to be commonly or uniquely expressed by pulmonary and extrapulmonary tissues was carried out using the RNA sequencing data from COVID-19 patients’ whole blood samples. The expression of these putative biomarkers was analyzed across different stages of disease severity, including healthy, asymptomatic, mild, and severe (subclassified into complicated, incremental complicated, hyperinflammatory complicated and critical) (17). A significant elevation in the expression of some of the proposed putative biomarkers was revealed in the blood of COVID-19 patients with severe hyperinflammatory disease presentation (**Figure 6A**). Some of these biomarkers include genes identified to be uniquely dysregulated in lung tissue (ABCE1, MR1, PARP14, and IFI16), liver tissue (NFASC), kidney tissue (LILRA5, S100A12, and LILRB5), and heart tissue (ASS1), in addition to genes commonly dysregulated across the pulmonary and extrapulmonary tissues (SOCS3 and TRIM56).

Moreover, qRT-PCR analysis of the whole RNA extracted from the blood serum of severe COVID-19 patients with pulmonary and extrapulmonary findings, confirmed the significant elevation in the gene expression of TRIM56 and SOCS3 in response to extrapulmonary dysfunction in severe COVID-19 patients (**Figure 6B**).

## Discussion

SARS-CoV-2 infection elicits aberrant transcriptional shifts in the expression of cytokines and immune mediators, resulting in an exacerbated systemic inflammation with detrimental consequences including multi-organ dysfunction (24). However, despite the existence of experimental evidence on SARS-CoV-2 infectivity of extrapulmonary tissues including liver, kidney, and heart tissues (4–6), the effect of the SARS-CoV-2 infection on their immune transcriptome is yet to be explored. Moreover, the contribution of these extrapulmonary tissues to the exacerbation of the cytokine storm in response to SARS-CoV-2 infection remains unclear. Therefore, in this study we aimed at investigating immune response shifts in pulmonary and extrapulmonary tissues (liver, kidney, and heart) in patients with COVID-19 using systems immunology analysis.

**Figure 7:**
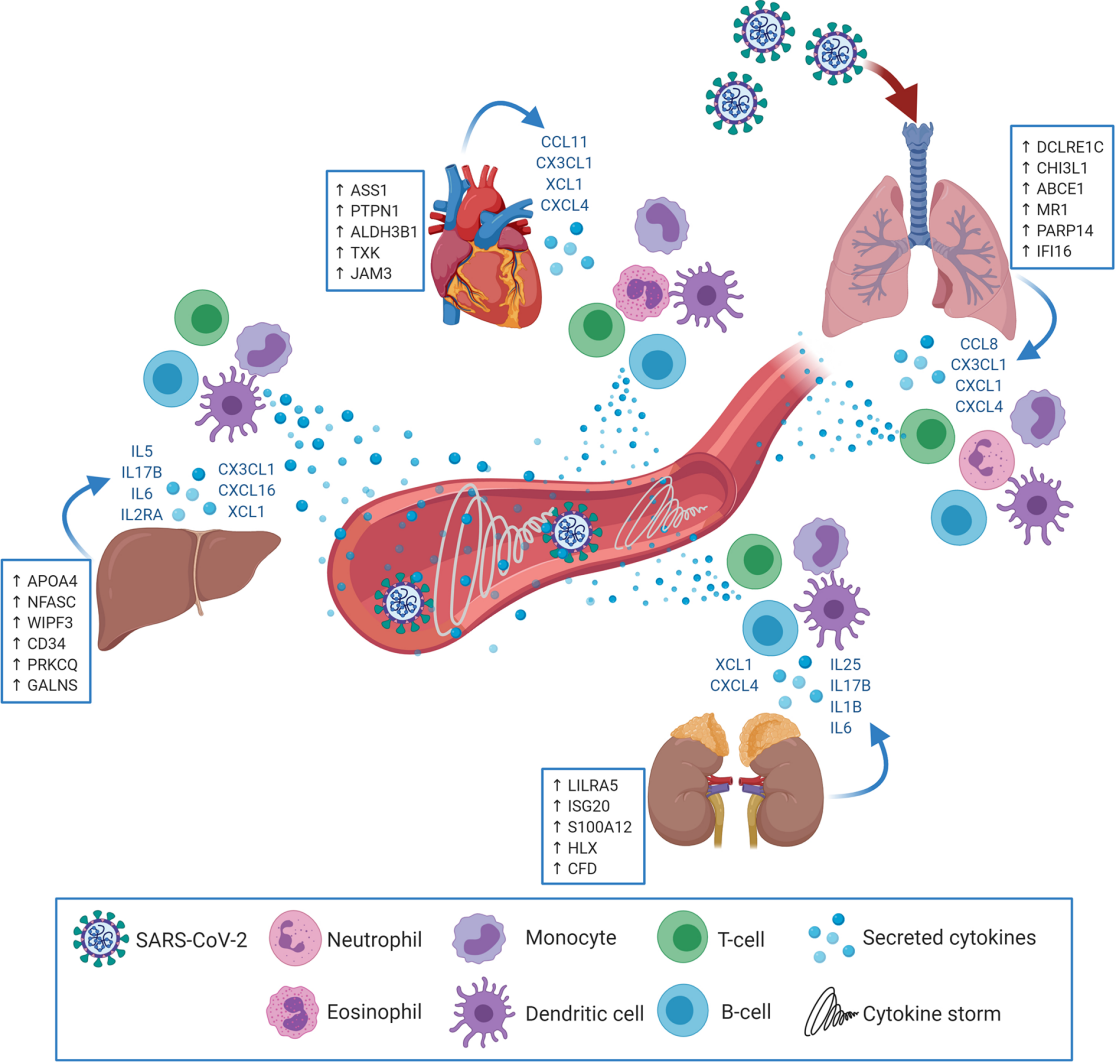
SARS-CoV-2 may target extrapulmonary tissues along pulmonary tissues thereby inducing transcriptional shifts in the expression of central immune regulators (examples in the blue boxes; putative biomarkers unique for each tissue). Consequently, pulmonary as well as extrapulmonary tissues may secrete cytokines and immune mediators (in blue font) resulting in the chemoattraction and activation of innate and adaptive immune cells (e.g. Neutrophils, eosinophils, monocytes, dendritic cells, T-cells and B-cells). Upon activation, the infiltrating immune cells begin to uncontrollably secrete additional cytokines and immune mediators which elicits the cytokine storm and system inflammation phenomena.

The results showed significant dysregulation of immune response genes in both pulmonary and extrapulmonary tissues; remarkably, the dysregulation in some extrapulmonary tissues (kidney) supersedes the dysregulation in pulmonary tissue. Moreover, this dysregulation of the immune response comprises the significant dysregulation of the innate and adaptive arms of the immune response in the upregulated and downregulated transcriptome of the four types of tissues. Although immune cells infiltration into some SARS-CoV-2 infected extrapulmonary tissues has been detected histologically (8, 10, 11), tissue-specific transcriptional shifts in immune response across different extrapulmonary tissues has not been explored before. For the first time we report that SARS-CoV-2 infection elicits immunomodulatory effects in extrapulmonary tissues in addition to pulmonary tissues (**Figure 7**).

Investigations on the pathogenesis of COVID-19 revealed a massive elevation of 14 cytokines levels in patient sera reflecting the cytokine storm and systemic inflammation (24). The analysis showed that out of these 14 cytokines, IL6 was significantly upregulated in liver and kidney tissues and IL2RA was significantly upregulated in liver tissue. More extended analysis of cytokines expression revealed the upregulation of neutrophil specific chemokine, CXCL1, in lung (25); the eosinophil chemoattractant, CCL11, in heart tissue (26); Monocytes chemoattractants in lung (*CXCL4, CCL8*, and *CX3CL1)*, liver, (*CX3CL1*), kidney (*CXCL4*), and heart (*CXCL4*, and *CX3CL1*) (27, 28); dendritic cell chemoattractants in lung (*CCL8* and *CX3CL1*), liver (*XCL1* and *CX3CL1*), kidney (*XCL1*), and heart (*XCL1* and *CX3CL1*) (29–33); and T-cells and B-cells chemotactic chemokines in lung (*CCL8, CXCL4* and *CX3CL1*), liver (*CXCL16*), kidney (*CXCL4* and *CXCL16*) and heart (*CXCL4* and *CX3CL1*) (29, 30, 32, 34). These findings suggest that the systemic cytokine storm is a product of the combined aberrant production of cytokines by pulmonary and extrapulmonary tissues.

The unique upregulation of CXCL1 in lung tissue in combination with gene set ontology analysis suggest that neutrophil-mediated inflammatory immune response is predominant in pulmonary tissue. This speculation is concordant with the clinical findings reporting the neutrophilic infiltration into patients’ pulmonary tissue in response to SARS-CoV-2 infection (61). Increased neutrophilic infiltration was proposed to contribute to the initiation of the cytokine storm, aggravation of immune response dysregulation, and potentially development of vascular thrombosis (61). Although CXCL1 was not reported to be upregulated in COVID-19 patient sera, it was highlighted as a significant element of upregulated transcriptome of SARS-CoV-2 infected normal human bronchial epithelial cells (62).

The unique upregulation of the eosinophil-specific chemoattractant, CCL11, suggests the potential activation of an eosinophil-mediated inflammatory response, which can be one of the mechanisms contributing to myocardial injury and heart failure in COVID-19 patients (63). However, as the upregulation of the eosinophilic response elements is unique to heart tissue, the lack of eosinophilic activation in lung tissues can potentially explain the lack of correlation between eosinophilic disorders (e.g. asthma) and SARS-CoV-2 as risk factors, on the contrary to other influenza virus infections (64).

Another important finding in this study is the significant enrichment of T-cell and B-cell chemoattractants in all analyzed tissues, which may explain the lymphocytic infiltrates into pulmonary and extrapulmonary tissues detected in COVID-19 patient autopsy samples (8, 10, 11). In this regards, it is plausible that the lymphopenia observed in COVID-19 patients (65) may result from increased lymphocytic recruitment to pulmonary and extrapulmonary tissues. Intriguingly, CKLF Like MARVEL Transmembrane Domain Containing 6 (CMTM6), a regulator and stabilizer of PD-L1 (66), was commonly upregulated across the four types of tissues. Through stabilizing and enhancing PD-L1 function, CMTM6 can contribute to excessive T-cell exhaustion, as observed in COVID-19 patients (67).

Histologic analysis revealed as well the infiltration of monocytes into lung, liver, kidney, and heart tissue in response to SARS-CoV-2 infection (68), which is consistent with the upregulation of monocyte chemoattractants in the four types of tissues in the transcriptome analysis. Remarkably, the consistent enrichment of transcripts implicated in immune response mediated by dendritic cells, monocytes, T-cell and B-cells suggests the activation of late stage innate immunity and adaptive immunity in both pulmonary and extrapulmonary tissues. However, the absence of significant Interferon-alpha/beta/gamma upregulation in the investigated pulmonary and extrapulmonary tissues confirms previous speculations on the shift of immune response polarization from a classic anti-viral response to a pathogenic inflammation that fails to safeguard against SARS-CoV-2 (69).

Functional clustering and pathway analysis of potential upstream regulators of the observed immune response dysregulation revealed NF-κB signaling as a potential upstream target of SARS-CoV-2 infection. NF-κB signaling was dysregulated in pulmonary and extrapulmonary tissues (liver, kidney, and heart) resulting a shift in the activation pattern of NF-κB signaling and consequent transcriptional shifts of NF-κB inducible genes including cytokines. Amongst the different elements dysregulated in the NF-κB signaling pathway across all the investigated tissues was the CBM complex (CARD-BCL10-MALT1), a molecular bridge that propagates extracellular immunomodulatory signals by regulating NF-κB signaling (70). Previous investigations on the molecular mechanisms underlying the pathogenesis of COVID-19 proposed NF-κB signaling as a potential mechanism underlying the initiation and polarization of the inflammatory response towards a pathogenic phenotype in pulmonary tissue (69). This study extended this theory to include extrapulmonary tissue as a target of SARS-CoV-2 immunomodulatory effect potentially through the dysregulation of NF-κB signaling.

Mucin family members including MUC4, MUC16, MUC20, MUC5AC, and MUC5B have been reported to be upregulated in bronchial club cells in response to SARS-CoV-2 infection (71). However, for the first time we report upregulation of multiple Mucin family members (e.g. *MUC3A, MUC4, MUC5B, MUC16*, and *MUC17*) in pulmonary and extrapulmonary tissues in response SARS-CoV-2 infection. Mucin family members, including MUC4, play important regulatory roles in proliferation, epithelial to mesenchymal transition (EMT), fibroblast to myofibroblast transition (FMT), tissue remodeling and fibrosis as observed in diseases such as idiopathic pulmonary fibrosis (IPF) and cancer (72–74).

Similarly, we report the novel finding on the significant upregulation of Heat Shock Protein 90 Alpha Family Class B Member 1 (HSP90AB1) was in the four types of pulmonary and extrapulmonary tissues. Beyond the role of HSP90AB1 in eliciting immune response and regulating viral infectivity (75), HSP90AB1 has been shown to promote tissue remodeling by stabilizing and enhancing TGF-β signaling (76). Moreover, HSP90AB1 promotes epithelial to mesenchymal transition by activating AKT and WNT signaling pathways through the WNT signaling receptor, LRP5 (77). Additionally, the infiltration of the immune effectors, including macrophages, into tissues in response to SARS-CoV-2 infection has been associated with tissue remodeling and fibrosis (68). Taken together, these results suggest that the exacerbated inflammation activates tissue remodeling genes which contributed to the observed pulmonary fibrosis and multi-organ dysfunction in COVID-19 patients.

Moreover, Lysophosphatidylcholine Acyltransferase 1 (LPCAT1), a commonly upregulated gene in SARS-CoV-2 infected pulmonary and extrapulmonary tissues, is an important regulator of the synthesis of potent inflammatory lipids such as platelet-activating factor and lysophosphatidylcholine (48). Besides its potential contribution to the modulation of the immune response and inflammation, the upregulation LPCAT1 could potentially link to the high venous and arterial thromboembolism incidence observed in severe cases of COVID-19 patients (~30.7%) (78).

The analysis further revealed potential tissue-specific immune signatures with uniquely upregulated immune response genes in each of the pulmonary and extrapulmonary tissues including *DCLRE1C, CHI3L1*, and *PARP14* in lung; *APOA4, NFASC, WIPF3*, and *CD34* in liver; *LILRA5, ISG20, S100A12*, and *HLX* in kidney; and *ASS1* and *PTPN1* in heart. The overexpression of some of these biomarkers was similarly observed in the blood serum of severe COVID-19 patients with extrapulmonary dysfunction (e.g. SOCS3 and TRIM56). The unique upregulation of these putative biomarkers offers a potential diagnostic approach to assess the inflammation of each of these extrapulmonary tissues. Since the exacerbation of the inflammation in liver, kidney and heart can potentially result in tissue dysfunction and potential organ failure, these biomarkers can be assessed for their potential to predict tissue dysfunction at early stages and therefore guide medical intervention. Therefore, further investigation on these biomarkers is required to assess their accessibility through liquid biopsies and their diagnostic and predictive potential in COVID-19 pathogenesis.

Whilst the main limitation is the lack of validation of the genes on patients’ tissue samples, nevertheless we feel that the systems immunology analysis carried out in this study have resulted in identifying unique set of genes that are linked to different tissues from severe COVID-19 patients warranting further studies to carry out the validation on different cohort of patients’ autopsies once they become available. In addition to address the validity of the results we carried out cross sample validation using a smaller number of samples (15 lung samples, 2 liver samples, 2 kidney samples and 3 heart samples) and the results were similar. For instance, the analysis revealed the unique upregulation of CXCL1 in lung, CCL11 in heart, and IL6 in liver and kidney as well as the common upregulation of mucin family genes and HSP90AB1 across the four types of tissues. Moreover, the identified unique set of genes were cross-validated using patients’ blood serum samples as well as publicly deposited RNA-seq data from COVID-19 patients whole blood samples. The cross-validation confirmed the upregulation of some of these genes in correlation with disease severity and extrapulmonary dysfunction.

## Data Availability Statement

Publicly available datasets were analyzed in this study. This data can be found here: COVID19 autopsy samples were retrieved from https://www.ncbi.nlm.nih.gov/geo/query/acc.cgi?acc=GSE150316, Gene Expression Omnibus (GEO), accession number GSE150316. Healthy autopsy samples were retrieved from https://www.ncbi.nlm.nih.gov/geo/query/acc.cgi?acc=GSE112356, Gene Expression Omnibus (GEO), accession number GSE112356. COVID-19 Whole blood RNA-seq dataset was retrieved from https://github.com/Systems-Immunology-IKMB/COVIDOMICs/tree/main/TF_enrichment/TF_enrichment_analysis-main/data.

## Ethics Statement

The studies involving human participants were reviewed and approved by Abu Dhabi Health COVID-19 Research Ethics Committee (DOH/DQD/2020/538), SEHA Research Ethics committee (SEHA-IRB-005) and Dubai Scientific Research Ethics Committee (DSREC-04/2020_09). The patients/participants provided their written informed consent to participate in this study.

## Author Contributions

SH, AH, MR, and RH were responsible for the conception, design, and development of the methodology. SH, AH, PB, AK, MR, and RH were responsible for the application of the methodology, the bioinformatics analysis and data interpretation. SH, AH, PB, HA, BM, AK, HB, RabH, QH, MR and RH were responsible for writing and reviewing the manuscript. RH, MR, QH, HB were responsible for the supervision of the study. All authors contributed to the article and approved the submitted version.

## Funding

This research has been financially supported by University of Sharjah COVID-19 grant (CoV19-0308) and Sharjah Research Academy (Grant code: MED001) and University of Sharjah (Grant code: 1901090254). HB and AK acknowledge funding by the Deutsche Forschungsgemeinschaft (DFG, German Research Foundation) under Germany’s Excellence Strategy – EXC 22167-390884018.

## Conflict of Interest

The authors declare that the research was conducted in the absence of any commercial or financial relationships that could be construed as a potential conflict of interest.
