# Supplementary material for: Systems Immunology Analysis Reveals the Contribution of Pulmonary and Extrapulmonary Tissues to the Immunopathogenesis of Severe COVID-19 Patients
Source: Front Immunol. 2021 Jun 28;12:595150. doi: 10.3389/fimmu.2021.595150 (PMC8273737; doi:10.3389/fimmu.2021.595150)
Supplement: Supplementary file 1 [file DataSheet_1.pdf]

Supplementary Material

1 Supplementary Tables

Table S1. Accession of Gene ontology sets retrieved from AmiGO 2 for the analysis of immune cell-specific gene enrichment

Accession	Term
**GO:0042119**	Neutrophil activation
**GO:0030593**	Neutrophil chemotaxis
**GO:0043307**	Eosinophil activation
**GO:0048245**	Eosinophil chemotaxis
**GO:0042117**	Monocyte activation
**GO:0002548**	Monocyte chemotaxis
**GO:0042116**	Macrophage activation
**GO:0048246**	Macrophage chemotaxis
**GO:0045576**	Mast cell activation
**GO:0002551**	Mast cell chemotaxis
**GO:0001773**	Myeloid dendritic cell activation
**GO:0002407**	Dendritic cell chemotaxis
**GO:0030101**	NK cell activation
**GO:0035747**	NK cell chemotaxis
**GO:0051132**	NK T cell activation
**GO:0042110**	T cell activation
**GO:0010818**	T cell Chemotaxis
**GO:0042113**	B cell activation
**GO:0035754**	B cell chemotaxis

Table S2. Details of primers used for qRT-PCR validation of gene expression

Gene symbol	Forward primer 5' to 3' sequence	Reverse primer 5' to 3' sequence	Amplicon Size (bp)
**SOCS3**	CATCTCTGTCGGAAGACCGTCA	GCATCGTACTGGTCCAGGAACT	98
**TRIM56**	ACCTACTGCCAAGACTGC	TGGTCTTGAAGGAGGCCAC	112
**18S**	TGACTCAACACGGGAAACC	TCGCTCCACCAACTAAGAAC	114

2 Supplementary Figures

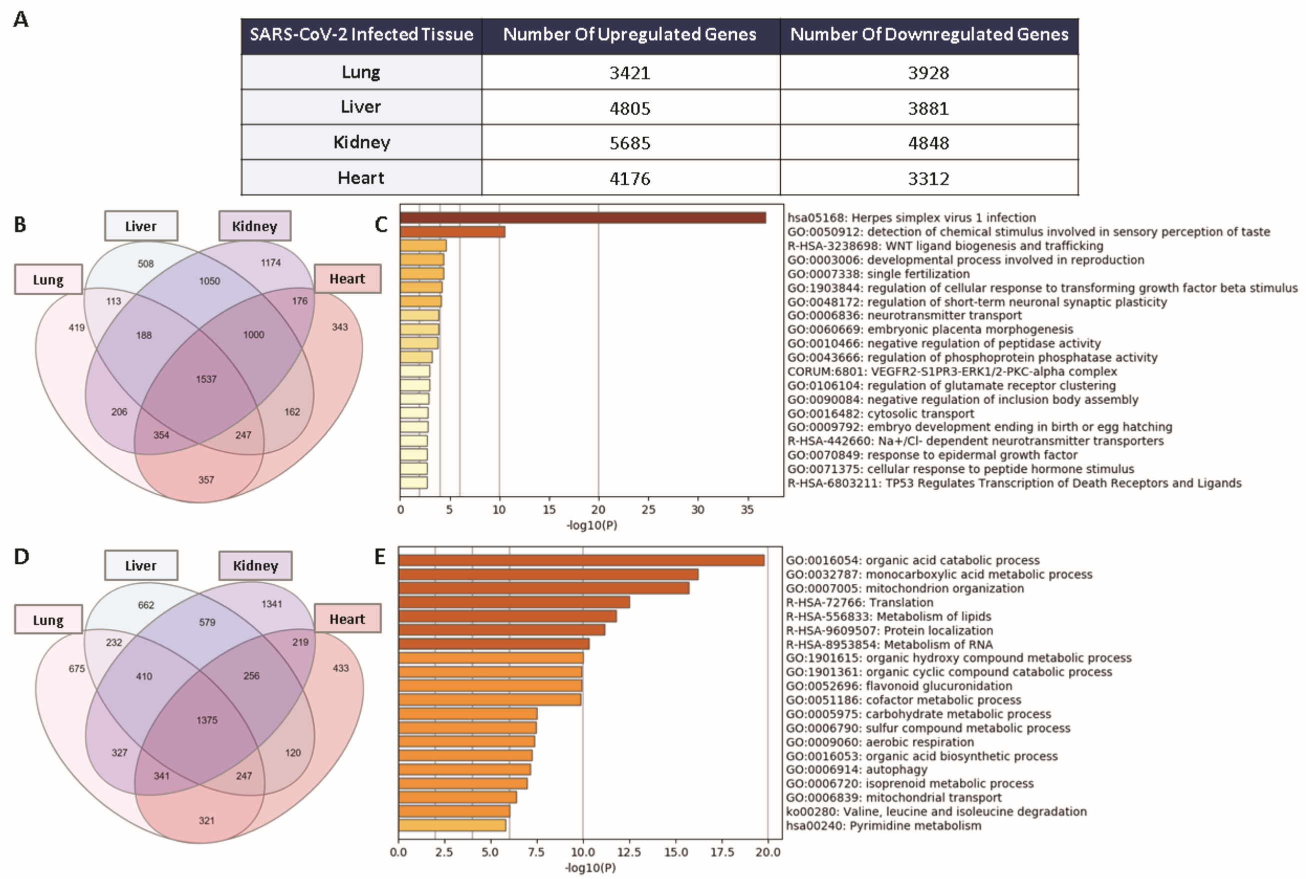

SARS-CoV-2 Infected Tissue	Number Of Upregulated Genes	Number Of Downregulated Genes
Lung	3421	3928
Liver	4805	3881
Kidney	5685	4848
Heart	4176	3312

Figure S1. (A) Count of differentially expressed genes from severe COVID-19 autopsy lung, liver, kidney, and heart tissue samples. Venn diagram representation of the overlap of significantly **(B)** upregulated and **(D)** downregulated genes amongst the four types of infected tissues. Functional clustering and pathway analysis of the commonly **(C)** upregulated and **(E)** downregulated genes across lung, liver, kidney, and heart tissues in response to SARS-CoV-2 infection.
